# 25-Hydroxyvitamin D_3 _1α-hydroxylase expression in breast cancer and use of non-1α-hydroxylated vitamin D analogue

**DOI:** 10.1186/bcr1332

**Published:** 2005-10-06

**Authors:** Ulrika Segersten, Pernille Kaae Holm, Peyman Björklund, Ola Hessman, Hans Nordgren, Lise Binderup, Göran Åkerström, Per Hellman, Gunnar Westin

**Affiliations:** 1 Department of Surgical Sciences, Endocrine Unit, Uppsala University Hospital, SE-751 85 Uppsala, Sweden; 2 Department of Biochemistry, Leo Pharmaceutical Products, DK-2750 Ballerup, Denmark

## Abstract

**Introduction:**

The cytochrome P450 mitochondrial enzyme 25-hydroxyvitamin D_3 _1α-hydroxylase (1α-hydroxylase) of renal tubule cells hydroxylates the major circulating form of vitamin D (25(OH)D_3_) to the active systemic hormone 1,25(OH)_2_D_3_. Local production of 1,25(OH)_2_D_3 _appears to occur also at other sites where 1α-hydroxylase is expressed for autocrine/paracrine regulation. To reduce risks of hypercalcemia during treatment with vitamin D, we have previously suggested use of non-1α-hydroxylated vitamin D analogues to target tissues where 1α-hydroxylase is expressed, including the parathyroid glands in secondary hyperparathyroidism. The present study was undertaken to examine expression of 1α-hydroxylase in breast cancer and to investigate whether a non-1α-hydroxylated vitamin D analogue displayed biological function. In addition, expression of the 25-hydroxyvitamin D_3 _24-hydroxylase (24-hydroxylase) and the vitamin D receptor (VDR) was investigated.

**Methods:**

The expression of 1α-hydroxylase, 24-hydroxylase and VDR was investigated in breast cancer specimens (n = 19) and normal breast tissues (n = 10) by immunohistochemistry and/or RT-PCR. Consecutive cryosections of 6 μm essentially free of immune cells were used in the analyses. The effect of vitamin D analogues on transcriptional activation was analyzed in transiently transfected MCF-7 breast cancer cells.

**Results:**

1α-hydroxylase protein was demonstrated in 79% and 100% of breast cancer specimens and normal breast, respectively. The overall relative mRNA levels of 1α-hydroxylase and 24-hydroxylase in normal breast compared to breast tumors were: 1α-hydroxylase, 1 ± 0.07 versus 0.7 ± 0.05, respectively (p < 0.001); 24-hydroxylase, 1 ± 0.08 verus 2.1 ± 0.2, respectively (p < 0.001). The VDR was expressed in 95% of the tumors as expected, with mRNA levels of 1 ± 0.09 and 1.4 ± 0.12 (p < 0.05) in breast cancer and normal breast, respectively. The ketoconazole-sensitive transcription activation potential of the non-1α-hydroxylated vitamin D analogue prodrug of EB1089 (EB1285) was demonstrated in MCF-7 cells, which express 1α-hydroxylase. The activity of EB1285 was about 20% of 1,25(OH)_2_D_3_.

**Conclusion:**

These results demonstrate nearly normal expression levels of 1α-hydroxylase, 24-hydroxylase and VDR in the majority of investigated breast cancer specimens. A non-1α-hydroxylated vitamin D analogue displayed activity in breast cancer cells. Such analogues may present future therapeutic options for proliferative disorders where 1α-hydroxylase is expressed.

## Introduction

Breast cancer is considered the most frequent malignancy of women in the western world. Surgery, radio-, chemo- and endocrine therapies are used in the treatment or prevention of this disease. During the past decade, the anticancer effects of 1,25(OH)_2_D_3 _and especially of the vitamin D analogue EB1089 have been well documented *in vitro *and *in vivo *[1,2]. Exposing the breast cancer cell line MCF-7 to 1,25(OH)_2_D_3 _or EB1089 elevates expression of the cell cycle restricting gene *p21*, promotes the dephosphosphorylated form of the retinoblastoma protein and keeps the cell in the G0-G1 stage of the cell cycle [3,4]. In addition, growth regression and pro-apoptotic effects of vitamin D analogues have been described in breast cancer cell lines as well as in animal models of breast cancer without or in combination with chemo- or endocrine therapy [5-12]. In a phase 1 clinical study, treatment with EB1089 resulted in stabilized disease in 4 out of 14 patients with advanced breast cancer [13].

The mitochondrial cytochrome P450 enzyme 25-hydroxyvitamin D_3 _1α-hydroxylase (1α-hydroxylase) is the key enzyme in systemic vitamin D activation [14,15]. Originally, 1α-hydroxylase was considered as a renal enzyme, but is now known to be expressed in many different tissues, such as the adrenal medulla, brain, pancreas, placenta [16], parathyroid gland [17], skin [14] and bone [18], with the possibility of local 1,25(OH)_2_D_3 _production and autocrine/paracrine regulation. Additionally, 1α-hydroxylase activity is not only present in normal tissue but also in colorectal [19-23] and prostate cancer [24-26]. Previously, we reported coincident increased expression of 1α-hydroxylase and reduced expression of 25-hydroxyvitamin D_3 _24-hydroxylase (24-hydroxylase) in the majority of investigated parathyroid adenomas and secondary hyperplastic glands from patients with primary- and secondary hyperparathyroidism, respectively [17,27]. Based on these results we suggested the use, in patients with hyperparathyroidism secondary to uremia, of non-1α-hydroxylated vitamin D analogues that may become hydroxylated locally in parathyroid cells to an active vitamin D receptor (VDR) binding compound with parathyroid hormone (PTH)suppressive and antiproliferative activities [27]. Here we have determined expression levels of 1α-hydroxylase, 24-hydroxylase and VDR in breast cancer cells and demonstrated the activity of a non-1α-hydroxylated vitamin D analogue.

## Materials and methods

### Tissue specimens

Nineteen fresh frozen breast cancer specimens were randomly selected from our tissue bank, stored at -70°C. Seventeen invasive ductal carcinoma and two invasive lobular carcinoma were examined. Also, 10 apparently normal breast tissue specimens from patients with breast cancer were included in the analysis. Consecutive cryosections essentially free of immune cells were used in the analyses. Informed consent and approval of an ethical committee was given.

### Immunohistochemistry

Acetone-fixed cryosections of 6 μm were immersed in 0.3% (v/v) H_2_O_2 _in methanol and then blocked with an avidin-biotin blocking kit (Vector Laboratories Inc., Burlingame, CA, USA) or normal goat serum. The specific 1α-hydroxylase sheep polyclonal peptide antiserum [17,28,29] or the VDR rabbit polyclonal peptide antiserum (sc-1008, Santa Cruz Biotechnology Inc., Santa Cruz, California, USA) were applied to the tissue sections and incubated at room temperature for 90 minutes using dilutions of 1:50 or 1:400, respectively. As secondary antibodies, the biotinylated donkey anti-sheep IgG (diluted 1/500) or a biotinylated goat anti-rabbit IgG (diluted1/200) were applied for 30 minutes, after which all sections were exposed to an avidin-biotin complex (Vector Laboratories Inc.). The immunoreaction was visualized with 3-amino-9-ethylcarbazole and the sections were counterstained with Mayer's hematoxylin. Tissue sections exposed to the 1α-hydroxylase antiserum or VDR antiserum preincubated with an excess of 1α-hydroxylase [17] or VDR (sc-1008P, Santa Cruz Biotechnology Inc.) immunizing peptides were used as controls. In addition, the 1α-hydroxylase staining procedure was also performed on acetone-fixed MCF-7 cells.

### Isolation of total RNA and cDNA synthesis

Total RNA was extracted from 10 consecutive frozen sections (6 μm) of the same tumor specimen (n = 19) and from normal (n = 10) breast tissues using TRIzol Reagent (Invitrogen Corp., Carlsbad, CA, USA) and treated with RQ1 DnaseI (Promega, Madison, WI, USA) and proteinase K. One μg RNA from each sample was reverse transcribed using a cDNA synthesis kit (Amersham Biosciences, Uppsala, Sweden).

### Semiquantitative RT-PCR analysis

Semiquantitative RT-PCR analysis was used for determination of relative mRNA expression levels of 1α-hydroxylase, 24-hydroxylase and VDR. 28S rRNA was used as internal standard [30]. The number of PCR cycles for each transcript was determined according to what gave measurable PCR products in the linear range of PCR amplification. The following mRNA-specific PCR primers were used: 1α-hydroxylase forward primer, 5'-GCT ACA CGA GCT GCA GGT GCA GGG C-3'; 1α-hydroxylase reverse primer, 5'-AGC GGG GCC AGG AGA CTG CGG AGC C-3'; 24-hydroxylase forward primer, 5'-GGC TTC TCC AGA AGA ATG CAG GGG ATG AAG-3'; 24-hydroxylase reverse primer, 5'-TGA GGC TCT TGT GCA GCT CGA CTG GAG TGA-3'; VDR forward primer, 5'-TGC CTG ACC CTG GAG ACT TTG ACC-3'; VDR reverse primer, 5'-CAT CAT GCC GAT GTC CAC ACA GCG-3'. For 28S rRNA the forward primer 5'-GTT CAC CCA CTA ATA GGG AAC GTG A-3' and reverse primer 5'-GGA TTC TGA CTT AGA GGC GTT CAG T-3' were used [30]. The sizes of the generated PCR products were 252 base pairs (bp) for 1α-hydroxylase [17], 117 bp for 24-hydroxylase, 242 bp for VDR and 212 bp for 28S rRNA. The PCR fragments displayed sequence identity to the published gene sequences for 1α-hydroxylase (GenBank: AB006987), for 24-hydroxylase (GenBank: NM_000782) and for VDR (GenBank: NM_000376). All the PCR reactions contained 2 μl, except 1 μl for 24-hydroxylase, of the cDNA-reaction, 0.2 mM dNTP, 1 × PCR-buffer, 1.5 mM MgCl_2_, 1.25 U platinum DNA-polymerase in a final volume of 50 μl. The primer concentrations were: 0.5 pmol/μl for 1α-hydroxylase; 0.2 pmol/μl for 24-hydroxylase; 0.4 pmol/μl for VDR; and 0.4 pmol/μl for 28S rRNA. In addition, the PCR reaction for 1α-hydroxylase contained 5% dimethylsulphoxide. Thermal cycler conditions for 1α-hydroxylase were: denaturation at 95°C for 1 minute, 38 cycles of denaturation at 95°C for 20 s, annealing at 64°C for 20 s, extension at 72°C for 20 s, followed by a final extension at 72°C for 7 minutes. For the other PCR products, the thermal cycler conditions were the same as for 1α-hydroxylase, except for 24-hydroxylase (40 cycles performed with annealing at 60°C for 20 s) and VDR (denaturation at 95°C for 2 minutes, 38 thermal cycles and annealing at 61°C for 20 s). For 28S rRNA, the thermal cycler conditions were denaturation at 95°C for 2 minutes and 20 cycles of denaturation at 95°C for 15 s, annealing at 66°C for 20 s followed by extension at 72°C for 10 s. All the PCR reactions were performed in a GeneAmp 9700 thermal cycler (Applied Biosystems, New Jersey, USA). After the indicated cycles of each amplification, 10 μl of each PCR reaction was separated on a 2.0% agarose gel with ethidium bromide. The intensity of each band was quantified by Molecular Analysis software (Bio-Rad Lab., Richmond, CA, USA). As negative controls, water was used instead of cDNA product to reveal false positive reactions.

### Transient transfection

MCF-7 cells were seeded at 2 × 10^5 ^cells per 35 mm dish on the day before transfection. A luciferase reporter gene plasmid (pMWM-30, MW Madsen, unpublished; 1 μg) with four copies of a DR3-type vitamin D response element from the rat atrial natriuretic factor promoter [31], an expression vector for VDR (pSG5-VDR; 0.5 μg) and the internal transfection control CMV-LacZ (0.1 μg) were cotransfected in triplicate using Fugene 6 (Roche Diagnostics Scandinavia AB, Bromma, Sweden). Vehicle (ethanol), vitamin D analogues or ketoconazole were added 4 h post-transfection at the indicated concentrations. Cells were harvested 24 h later, and luciferase and β-galactosidase activities were determined luminometrically.

### Statistical analysis

Unpaired Student's *t*-test was used and data were calculated with Stat View 5.0 (SAS Institute Inc., Cary, NC, USA). Values are presented as mean ± standard error of the mean.

## Results

### Expression of 1α-hydroxylase, 24-hydroxylase and VDR in breast cancer

**Figure 1:**
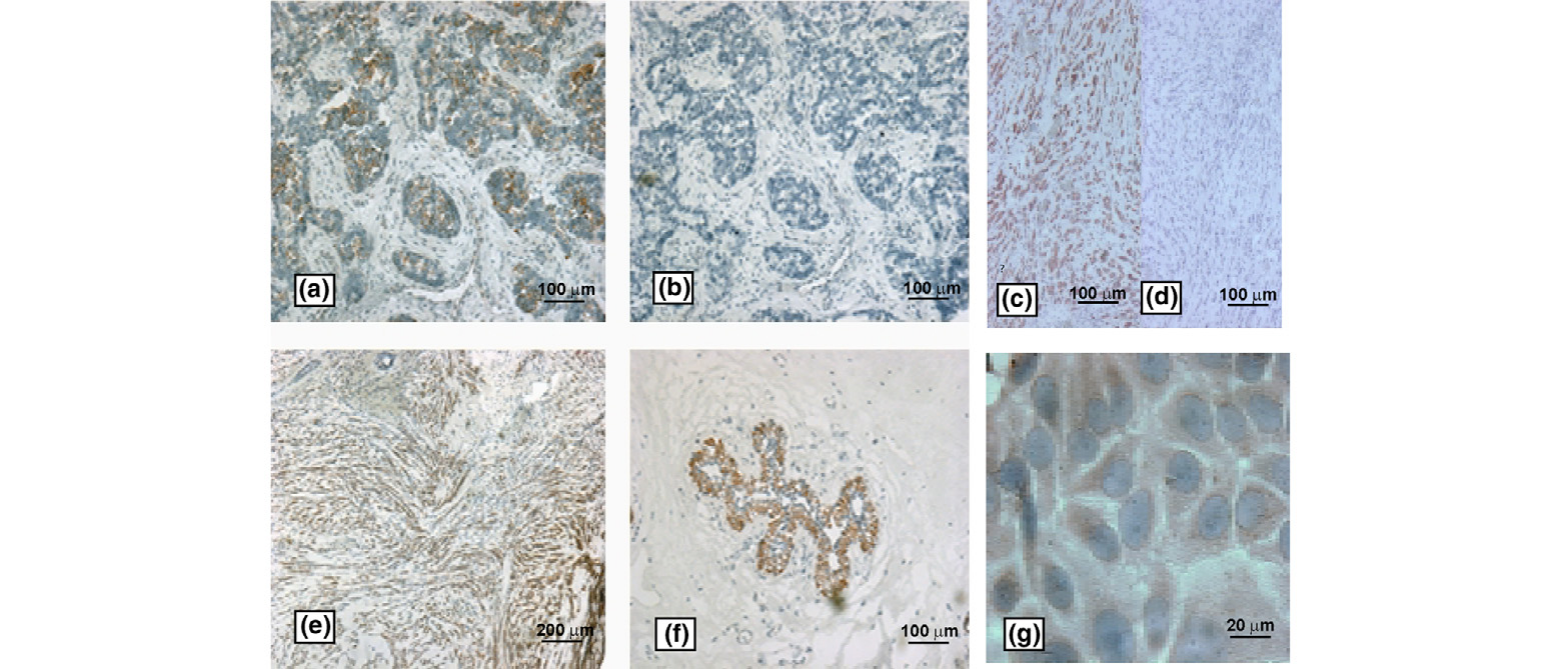
Expression of 1α-hydroxylase in breast cancer. Immunohistochemical analysis of 1α-hydroxylase expression in **(a) **ductal breast cancer, **(e) **lobular breast cancer, **(f) **normal breast and **(g) **MCF-7 cells. **(c) **Expression of VDR in lobular breast cancer. Control sections were exposed to **(b) **1α-hydroxylase and **(d) **VDR peptide antisera pre-incubated with immunizing peptide.

Immunohistochemical analysis of 1α-hydroxylase expression showed distinct cytoplasmic specific immunoreactivity for 14 of the 17 analyzed ductal breast cancer specimens, for one of two lobular specimens and for all 10 normal breast tissues (Fig. 1a,b,e,f). All three non-staining ductal cancers showed specific immunoreactivity in the benign part of the specimen. For both tumor and normal tissues, areas of variable size showed intensely stained cells mixed with weakly or non-staining cells. 1α-hydroxylase was also found to be expressed in the MCF-7 breast cancer cell line (Fig. 1g). In general, specific VDR immunoreactivity appeared similar to 1α-hydroxylase but also with weak nuclear staining (Fig. 1c,d). All analyzed specimens, except for one ductal breast cancer and one normal breast, stained for VDR.

**Figure 2:**
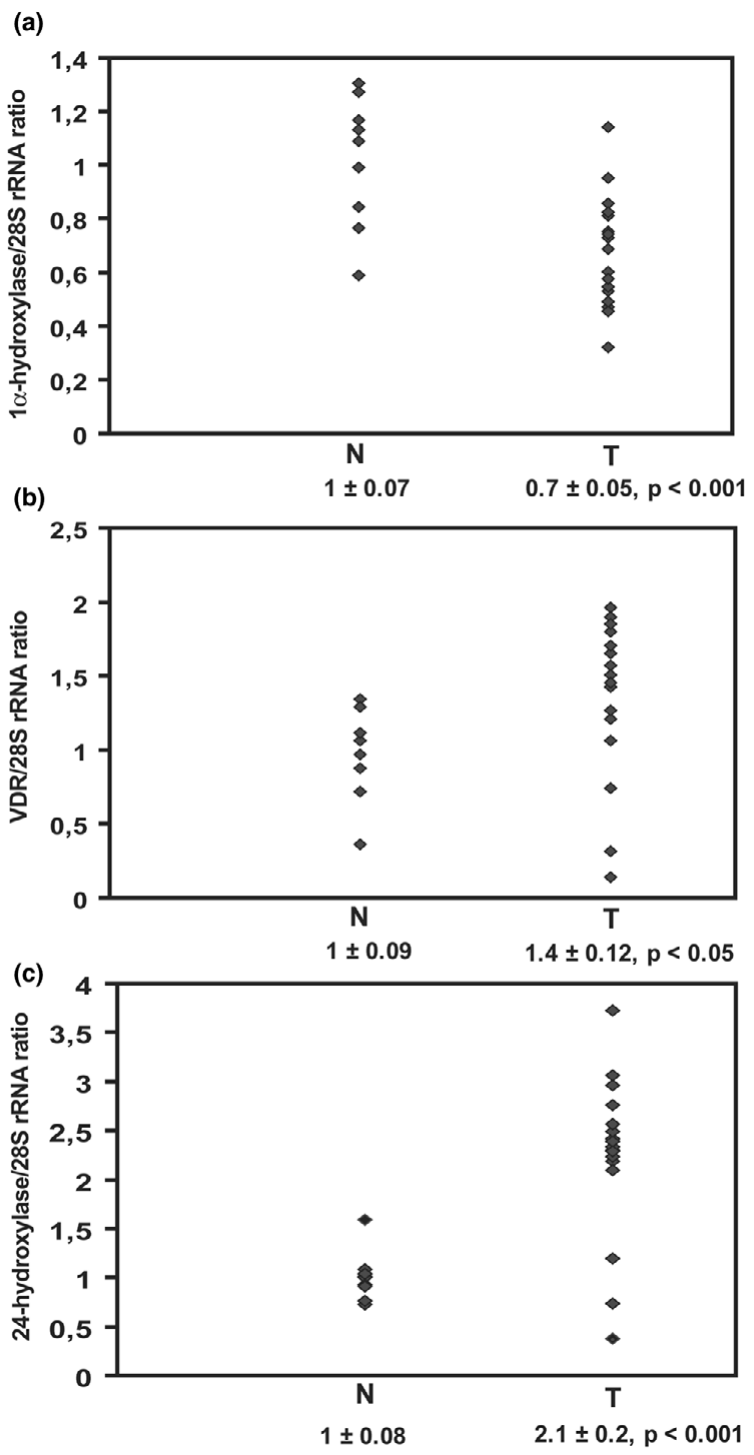
**(a) **Determination of 1α-hydroxylase, **(b) **VDR and **(c) **24-hydroxylase mRNA expression in normal (N) breast tissue specimens (n = 10) and breast cancer (T) specimens (n = 19) by semiquantitative RT-PCR. The expression was related to the 28S rRNA level in each sample. The results are presented as mean ± standard error of the mean. and comparisons between the group means were analyzed by Student's *t*-test.

Next, we determined the mRNA expression levels for 1α-hydroxylase, VDR and 24-hydroxylase in relation to 28S rRNA. We chose to use 28S rRNA as a comparative control because glyceraldehyde-3-phosphate dehydrogenase is not recommended in breast cancer [32]. Total RNA was isolated from frozen sections of the same tumor (n = 19) and normal (n = 10) breast tissues analyzed above. The results of the semi-quantitative RT-PCR analysis are shown in Fig. 2. In comparison to the normal breast tissues, the 1α-hydroxylase/28S rRNA ratio (Fig. 2a) was somewhat lower (1 ± 0.07 versus 0.7 ± 0.05, p < 0.001) and the VDR/28S rRNA ratio (Fig. 2B) somewhat higher (1 ± 0.09 versus 1.4 ± 0.12, p < 0.05) in the breast tumor specimens. Expression of 24-hydroxylase (Fig. 2c) was two-fold higher in the tumors compared to normal tissues (2.1 ± 0.2 versus 1 ± 0.08, p < 0.001). These results were consistent with the immunostainings of 1α-hydroxylase and VDR. The three tissue specimens (two tumors, one normal) with no detected staining at all showed the lowest mRNA expression levels for 1α-hydroxylase and VDR, respectively.

### A non-1α-hydroxylated vitamin D analogue activates transcription in MCF-7 cells

**Figure 3:**
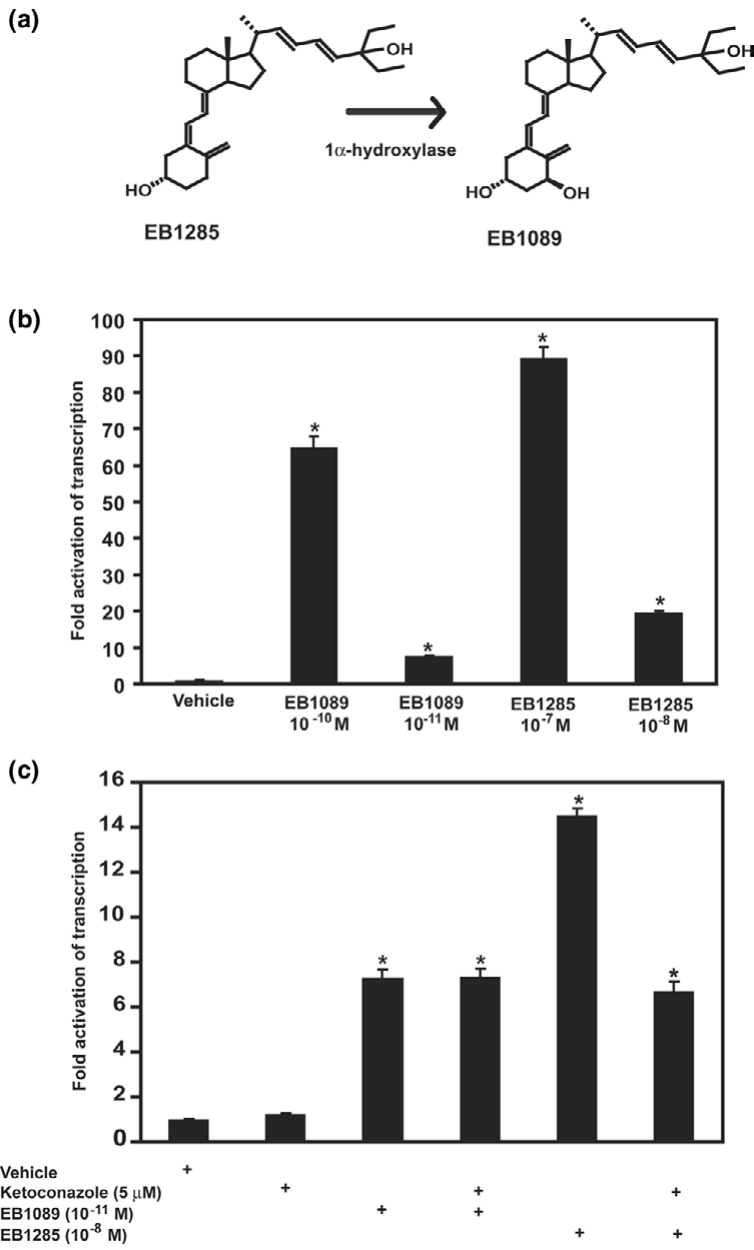
Transcription activity of EB1285 in MCF-7 cells. **(a) **The chemical structure of the vitamin D analogue EB1285 before and after (EB1089) 1α-hydroxylation. **(b) **VDR-directed transcription activity of EB1089 or EB1285 in transfected MCF-7 cells. **(c) **Activation of transcription in the presence of the cytochrome P450 inhibitor ketoconazole. Asterisks represent p < 0.001.

We have recently suggested that inactive non-1α-hydroxylated vitamin D analogues, with inherent low hypercalcemic and hyperphosphatemic toxicity, could potentially become 1α-hydroxylated locally in 1α-hydroxylase expressing cells and, thereafter, execute biological functions by binding to the VDR [27]. To test this idea experimentally in MCF-7 cells, we chose the non-1α-hydroxylated form of EB1089 (Fig. 3a). This vitamin D analogue (EB1285) is stable, has very low affinity for the VDR and shows low calcemic effects [33] compared to 1,25(OH)_2_D_3 _in normal rats (Kaae Holm, unpublished). To investigate biological activity of the non-1α-hydroxylated vitamin D analogue EB1285, we performed a transient expression analysis using a vitamin D response element-reporter gene construct co-transfected together with a VDR expression vector and an internal transfection control plasmid. EB1285 activated transcription 89-fold from the VDRE reporter gene in transfected MCF-7 cells at a concentration of 100 nM and 20-fold at 10 nM (Fig. 3b). EB1089 displayed high potency, as expected. Furthermore, in the presence of the cytochrome P450 inhibitor ketoconazole, activation by EB1285 was reduced by 50% (Fig. 3c), which would be expected [34] if transcription activation was dependent on 1α-hydroxylase enzymatic activity. EB1089 activated transcription to the same extent regardless of ketoconazole addition (Fig. 3c), possibly due to its resistance to inactivation by 24-hydroxylase activity [35,36]. The transcriptional activity of EB1285 and EB1089, compared to 1,25(OH)_2_D_3 _(EC_50 _= 1.0), was 0.2 and 105, respectively (data not shown). The results support the idea that an inactive non-1α-hydroxylated vitamin D analogue can become hydroxylated and activated in cell culture (MCF-7), although with low relative efficiency as shown here for EB1285.

## Discussion

In the present study, we have demonstrated 1α-hydroxylase protein expression in 15 out of 19 (79%) analyzed breast cancer specimens, in 10 apparently normal breast biopsies from breast cancer patients and also in MCF-7 cells. The observed somewhat reduced overall 1α-hydroxylase mRNA expression level as well as expression of VDR protein and mRNA in the tumors were apparently consistent with the immunohistochemical results, also indicating that representative mRNA was isolated. Of the analyzed tumors, 95% stained for VDR, in agreement with the 80% to 90% observed in earlier studies [37,38].

The 24-hydroxylase mRNA level was overall two-fold higher in breast carcinoma as compared to normal tissue. Notably, the *CYP24 *gene has been described as a breast candidate oncogene [39].

Previous studies have demonstrated 1α-hydroxylation of the prohormone 25(OH)D_3 _and inhibition of cell proliferation in cultured prostate cancer cells expressing 1α-hydroxylase [24,26]. 1α-hydroxylase is also expressed and active in colorectal cancer [20-23] and in ovarian cancer [40]. The non-calcemic prohormone 25(OH)D_3_, which exhibits very low activity *in vitro *and *in vivo *in the absence of 1α-hydroxylase [41,42], has been considered a future preventive and/or therapeutic option. A problem is rapid 24-hydroxylation and subsequent degradation of 25(OH)D_3 _and of locally synthesized 1,25(OH)_2_D_3_. Use of more specific 24-hydroxylase inhibitors [43] than liarozole and ketoconazole [34,44] may present future therapeutic options. We reasoned that an alternative to 25(OH)D_3 _could be a non-1α-hydroxylated vitamin D analogue [27], with a relative resistance to 24-hydroxylation by the 24-hydroxylase. The activity of 1α-hydroxylase in the kidney is tightly regulated by PTH and 1,25(OH)_2_D_3 _and even large increases in serum 25(OH)D_3 _will not produce hypercalcemia [25]. Similarily, local production of a VDR binding analogue by hydroxylation would not be expected to cause the systemic effect of hypercalcemia. Here we have shown that the non-1α-hydroxylated prodrug of EB1089 (EB1285) could activate transcription in MCF-7 cells, which express the 1α-hydroxylase enzyme. The activation of transcription was ketoconazole-sensitive, strongly suggesting that the observed effect was due to 1α-hydroxylation. Thus, a vitamin D analogue could constitute a substrate for the 1α-hydroxylase enzyme; however, EB1285 exhibited low transcription activation potential compared to EB1089 in MCF-7 cells. This may indicate low 1α-hydroxylase enzyme activity in the cells and/or possibly inefficient hydroxylation due to steric hindrance between substrate and enzyme. Design of novel non-1α-hydroxylated vitamin D analogues for the prevention or treatment of proliferative disorders in which 1α-hydroxylase is expressed or induced is warranted.

## Conclusion

The findings imply that a vitamin D analogue could constitute a substrate for the 1α-hydroxylase enzyme and that more efficient non-1α-hydroxylated analogues should be considered for treatment of human diseases in which 1α-hydroxylase is expressed, such as breast cancer and secondary hyperparathyroidism [17,27].

## Abbreviations

1α-hydroxylase = 25-hydroxyvitamin D_3 _1α-hydroxylase; 24-hydroxylase = 25-hydroxyvitamin D_3 _24-hydroxylase; bp = base pairs; PTH = parathyroid hormone; VDR = vitamin D receptor

## Competing interests

US obtained funding and salary from Leo Pharmaceutical Products.

## Authors' contributions

US and PB carried out the experimental studies, interpretation, performed the statisical analysis, and helped to draft the manuscript. PKH performed some experiments and LB provided EB1285 and EB1089. OH and HN collected and analyzed the clinical data. GÅ and PH helped to draft the manuscript. GW conceived of the study, participated in its design and coordination, performed some experiments, and drafted the manuscript.
